# Genome sizes of animal RNA viruses reflect phylogenetic constraints

**DOI:** 10.1093/ve/veaf005

**Published:** 2025-01-24

**Authors:** Kosuke Takada, Edward C Holmes

**Affiliations:** School of Medical Sciences, The University of Sydney, Sydney, NSW 2006, Australia; Department of Molecular Virology, Research Institute for Microbial Diseases, Osaka University, Osaka 565-0871, Japan; School of Medical Sciences, The University of Sydney, Sydney, NSW 2006, Australia

**Keywords:** Genome size, Nidovirales, Segmentation, Evolution, RNA virus

## Abstract

Animal genomes are characterized by extensive variation in size. RNA viruses similarly exhibit substantial genomic diversity, with genome lengths ranging from 1.7 to ∼64 kb. Despite the myriad of novel viruses discovered by metagenomics, we know little of the factors that shape the evolution of the genome size in RNA viruses. We analyzed the variation in genome sizes across orders and families of animal RNA viruses. We found that RNA viruses can have highly variable genome sizes within and among orders, with the *Nidovirales* (including the *Coronaviridae*) exhibiting both significantly larger genomes and a greater range of genome sizes than other orders. In the *Bunyavirales, Amarillovirales, Nidovirales*, and *Picornavirales*, the genome sizes of invertebrate-associated RNA viruses were significantly larger than those that infect vertebrates, in contrast to their animal hosts in which vertebrates commonly have larger genomes than invertebrates. However, in the *Mononegavirales*, vertebrate viruses were significantly larger than those viruses associated with invertebrates. There were similarly complex associations between genome size and patterns of genome segmentation. In the *Bunyavirales, Reovirales*, and *Nidovirales*, viruses with segmented genomes, or that possessed a large number of segments, had significantly larger genome sizes than viruses with nonsegmented genomes or a small number of segments, while in *Articulavirales*, there were no significant differences in genome size among viruses possessing any number of genome segments. More broadly, our analysis revealed that taxonomic position (i.e. RNA virus order) had a greater impact on genome size than whether viruses infected vertebrates or invertebrates or their pattern of genome segmentation. Hence, the phylogenetic constraints on genome size are of sufficient magnitude to shape some other aspects of virus evolution.

## Introduction

Animal genomes are characterized by extensive variation in size. In general, vertebrate genomes are larger and more complex than those of invertebrates (Gregory 2005), varying from around 400 million base pairs (Mb) in some fish (e.g. *Takifugu rubripes*) (Aparicio et al. 2002, Gregory 2024) to billions of base pairs (Gb) in mammals and birds (Venkatesh et al. 2000, Gregory 2005). The largest vertebrate genome reported to date is ∼130 Gb in the lungfish *Protopterus aethiopicus*, while genomes of ∼117 Gb are found in the amphibians *Necturus lewisi* and *Necturus punctatus*. Repetitive DNA sequences, such as transposable elements (i.e. DNA transposons and retrotransposons), are a major contributor to the large genome sizes of some vertebrate species and associated with increases in genome size and complexity (Kidwell 2002, Biscotti et al. 2015, Canapa et al. 2015). In contrast, invertebrate genome sizes may be only ∼20 Mb in the nematode *Pratylenchus coffeae* and ∼180 Mb in the arthropod *Drosophila melanogaster*, but over 1 Gb for certain insects and marine invertebrates (Canapa et al. 2015, Gregory 2024). It is important to note, however, that proportionally far fewer genomes have been sequenced from invertebrates than vertebrates. Despite these sampling biases, it is clear that there are marked differences in the size of animal genomes.

Animals (*Metazoa*) evolved from a single-celled ancestor more than 635 million years ago, an evolutionary transition that was associated with a dramatic increase in phenotypic diversity, including the rapid diversification of multiple animal phyla (Davidson and Erwin 2009, Dos Reis et al. 2015, Ros-Rocher et al. 2021). A key evolutionary innovation associated with the later rise of the vertebrates was the adaptive immune system, first appearing in jawed fish about 500 million years ago and leading to major differences in how vertebrates respond to viral infections compared to their invertebrate ancestors (Flajnik and Kasahara 2010). In particular, while the invertebrate immune response is innate and usually stereotypic, that of vertebrates is more complex and has a strong adaptive capacity, allowing for a more rapid and targeted immune response when pathogens are repeatedly encountered (Du Pasquier 2001, Boehm 2012). It might also be expected that such a major transition in host evolution, concordant with the evolution of the vertebrates, would impact the evolution of the viruses that infect them, including the size and complexity of their genomes. For example, it has been hypothesized that the evolution of the adaptive immune system would select for smaller RNA virus genomes as this would theoretically result in fewer immune targets (Harvey and Holmes 2022).

Viruses are similarly characterized by considerable genomic diversity. Genome sizes in RNA viruses range from only ∼1.7 kb (Hepatitis delta virus, family *Kolmioviridae*) to more than 64 kb (members of the order *Nidovirales*) (Gorbalenya et al. 2006, Neuman et al. 2024). Although the metagenomic sequencing of diverse animal taxa in recent years has led to a marked increase in our knowledge of genome sizes in RNA viruses compared to only a decade ago (Weaver et al. 2016), particularly in the case of the *Nidovirales* (Neuman et al. 2024), they are usually still far smaller than those of DNA viruses. Indeed, the genome sizes of DNA viruses range from ∼1.8 kb for the (single-stranded) circoviruses to ∼2.5 Mb for the pandoraviruses (Philippe et al. 2013).

The constraint on the maximum length of RNA virus genomes has commonly been associated with the low fidelity of their replication process, central to which is an RNA-dependent RNA polymerase (RdRp) that lacks proofreading ability and hence results in a high mutation rate per genome replication (Holmes 2009). Indeed, there is a broad inverse correlation between genome size and mutation rate (Duffy et al. 2008, Sanjuan et al. 2010), such that an increase in virus genome size requires a reduction in mutation rate per nucleotide to limit the accumulation of deleterious mutations that result in major fitness losses (Holmes 2003). An exception that goes some way to proving this rule is the *Coronaviridae*, again members of order *Nidovirales*, that possesses a 3'-to-5' exoribonuclease (ExoN) protein (i.e. N-terminal domain of nonstructural protein 14) that provides a form of RNA proofreading that reduces error rates and in so doing facilitates the evolution of longer genomes (Gorbalenya et al. 2006, Eckerle et al. 2010, Liao et al. 2023). Although it is unclear whether similar error-correction mechanisms exist in other RNA viruses with large genome sizes, it is noteworthy that a 39.8-kb virus was recently identified within the *Flaviviridae* (order *Amarillovirales*) that does not encode an exoribonuclease or known error-correction protein (Petrone et al. 2024).

It may also be that genome sizes of RNA viruses differ according to their type of genome organization. In particular, it has been suggested that segmented RNA viruses may have larger genomes than nonsegmented (i.e. single segment) RNA viruses because this reduces mutation accumulation by facilitating the more efficient selective removal of deleterious mutations through genomic reassortment (Pressing and Reanney 1984, Chao 1990). Indeed, an early study showed that the average size of segmented RNA virus genomes was slightly larger, at ∼11 kb, than that of nonsegmented RNA viruses (9 kb, although excluding members of the *Coronaviridae* that possess ExoN domains) (Holmes 2009). Currently, the largest reported segmented RNA viruses are some bisegmented members of the order *Nidovirales* with genomes that exceed 36 kb (Neuman et al. 2024). To date, however, there has been no systematic assessment of the relationship between the patterns of genome segmentation and genome size.

Despite the huge expansion in the known virosphere that has resulted from the development of metagenomic sequencing, we currently have little understanding of the factors that shape the evolution of the genome size of RNA viruses, particularly if they differ fundamentally between hosts—vertebrates and invertebrates—that themselves differ in genome size and complexity. To better understand the factors responsible for the range of genome sizes of RNA viruses, we performed a detailed analysis of the variation in genome sizes across different orders and families of RNA viruses and examined the differences between nonsegmented and segmented viruses and those that infect vertebrates or invertebrates.

## Materials and methods

### Sequence collection

Complete genome sequences of RNA viruses were obtained from the Virus Genome Resource (https://www.ncbi.nlm.nih.gov/genomes/GenomesGroup.cgi?taxid=10239&sort=taxonomy) in May 2024. Partial sequences and those without host and viral order/family annotation were excluded. We only considered animal RNA viruses (excluding those from plants and other eukaryotes) as this enabled us to assess how the major innovations associated with the origin of the vertebrates, particularly the evolution of adaptive immunity, may have impacted the diversity and structure of the viruses infecting them. We supplemented these data with viral sequences collated from the National Center for Biotechnology Information/GenBank database and a relevant publication (Neuman et al. 2024). Accordingly, the total number of RNA viruses with complete genomes analyzed in this study was 1467, representing 18 orders (including 58 families) of RNA viruses (Supplementary Table 1). The genome sizes of viruses with segmented genomes were calculated by summing the sizes of each genome segment. For analyses of the RdRp gene alone, we added 92 viruses, comprising 18 from the *Articulavirales*, 41 from the *Bunyavirales*, 12 from the *Mononegavirales*, and 21 from the *Jingchuvirales* (Supplementary Table 2). The length of the RdRp genes of viruses in the *Negarnaviricota* and *Duplornaviricota* was measured from the start to the stop codon (Supplementary Tables 1 and 2). In the case of the *Articulavirales* which have a complex of replication proteins, two methods were used to calculate the size of the RdRp: (i) summing the sizes of three genes that encode polymerase basic protein 1 (PB1), polymerase basic protein 2 (PB2), and polymerase acidic protein and (ii) utilizing the PB1 gene only (Supplementary Fig. 7).

**Table 1.** Comparison of genome sizes of animal viruses within orders of RNA viruses.

	Virus order (-*virales*)																	
	*Articula*	*Bunya*	*Mononega*	*Jingchu*	*Mu*	*Goujian*	*Reo*	*Ghabri*	*Hepeli*	*Martelli*	*Amarillo*	*Nodamu*	*Tymo*	*Toli*	*Durna*	*Nido*	*Picorna*	*Stella*
Number of viruses	24	**318**	308	8	7	2	70	17	8	26	145	19	3	**1**	6	157	301	47
Minimum	10 323	8108	6275	7977	7366	7768	6677	4647	5329	11 245	8568	4294	6169	6155	**3657** ^a^	**12 704**	6434	5547
10% percentile	10 425	10 463	10 712	7977	7366	7768	**17 935**	5493	5329	11 369	9355	4322	6169	6155	**3657**	15 296	7213	6097
25% percentile	10 781	11 364	11 215	9853	7380	7768	18 910	6121	6690	11 434	10 025	4505	6169	6155	**3941**	**20 227**	7439	6301
Median	12 337	12 193	12 399	11 020	7661	7973	19 917	6688	7077	11 574	10 755	5877	6258	6155	**4251**	**27 464**	8013	6543
75% percentile	13 489	12 603	15 303	11 852	7812	8178	23 606	7706	7670	11 692	11 020	5921	6532	6155	**4322**	**30 134**	9310	6712
90% percentile	13 609	16 785	16 277	12 996	8238	8178	25 137	8644	9762	11 906	12 631	6281	6532	6155	**4396**	**32 401**	10 179	7080
Maximum	14 452	25 142	20 148	12 996	8238	8178	29 174	10 316	9762	12 096	39 834	6410	6532	6155	**4396**	**64 336** ^b^	12 333	7722
Range	4129	17 034	13 873	5019	872	410	22 497	5669	4433	851	31 266	2116	363	**0**	739	**51 632**	5899	2175
Mean	12 261	12 514	13 108	10 846	7679	7973	21 171	6924	7224	11 590	11 546	5267	6320	6155	**4148**	**26 178**	8412	6548
SD	1 286	2347	2595	1528	308.2	289.9	3409	1305	1250	203.3	3865	814.5	189.2	**0**	268.1	**7457**	1231	410.3
Lower 95% confidence interval (CI) of mean	11 718	12 255	12 817	9569	7394	5368	20 358	6253	6179	11 508	10 912	4875	5850	N.A.	**3866**	**25 002**	8273	6428
Upper 95% CI of mean	12 804	12 773	13 399	12 123	7964	10 578	21 984	7594	8269	11 672	12 181	5660	6790	N.A.	**4429**	**27 354**	8552	6669
Skewness	−0.2183	1.86	0.4343	−0.7597	0.9143	N.A.	−0.6449	0.8546	0.9204	0.6565	**4.094**	−0.04316	1.311	N.A.	**−1.535**	0.9727	0.8723	0.3862
Kurtosis	−1.317	4.342	−0.2245	1.019	0.6519	N.A.	3.554	1.703	2.772	0.1464	**21.67**	**−2.038**	N.A.	N.A.	2.249	4.473	0.03276	1.115

Bold numbers indicate the maximum and minimum values within each row. N.A., not applicable due to insufficient sample size.

a The smallest virus genome size of all the viruses analyzed in this study (i.e. Raphanus sativus cryptic virus 1; NC_008190, NC_008191).

b The largest virus genome size of all the viruses analyzed in this study [i.e. Crassostrea gigas nidovirus; Neuman et al. (2024)].

### Statistical analysis

Values of skewness and kurtosis were computed to assess the asymmetry and sharpness of the distribution. To calculate skewness and kurtosis for the sample size $n$, we subtracted the mean value $\bar{\mathcal{X}}$ from each data ${\mathcal{X}_i}$ and standardized the result by dividing by the standard deviation $s$. For small samples, bias corrections were applied.

Skewness is adjusted using a correction factor $\left( {\frac{{\sqrt {n\left( {n - 1} \right)} }}{{n - 2}}} \right)$:


$${{Skewness}} = \left( {\frac{{\sqrt {n\left( {n - 1} \right)} }}{{n - 2}}} \right)\frac{1}{n}\mathop \sum \limits_{i = 1}^n{\left( {\frac{{{\mathcal{X}_i} - \bar{\mathcal{X}}}}{s}} \right)^3}.$$

Kurtosis is calculated as


$${{Kurtosis}} = \frac{1}{{\left( {n - 1} \right)}}\mathop \sum \limits_{i = 1}^n{\left( {\frac{{{\mathcal{X}_i} - \bar{\mathcal{X}}}}{s}} \right)^4} - 3.$$

A homogeneity of variance test was performed by using mean-based Levene’s test (Draper and Hunter 1969), which is the one-way analysis of variance *F*-test on $\left| {{\mathcal{X}_{ij}} - {{\bar{\mathcal{X}}}_i}} \right|$, the absolute deviations of the ${\mathcal{X}_{ij}}$ from their group mean ${\bar{\mathcal{X}}_i}$. Mean values were compared by using a one-way analysis of variance (ANOVA), followed by Tukey’s multiple comparison test. A *P*-value < .05 denoted statistical significance.

We used linear regression to determine whether there was a correlation between viral genome size and the size of the gene encoding the RdRp. Accordingly, linear regression graphs were obtained using Graph Prism 10 software (version 10.3.1). The statistical significance of each variable was evaluated with a *P*-value (*P *< .05 was considered significant), and the goodness of fit of the overall model was indicated by *R*^2^. To compare the genome size or size of the gene encoding the RdRp, the mean values within each viral order were compared by using a one-way ANOVA, followed by Šídák’s multiple comparisons test, with *P*-value < .05 again denoting statistical significance. All statistical analyses were performed using Graph Prism 10 software (version 10.3.1) and in-house python scripts.

### Random Forest regression model

A Random Forest regression was employed to model the relationship between the features and the target variable, in this case genome size. Various categorical features, including host (i.e. invertebrate and/or vertebrate), viral order (18 viral orders), and genome segmentation (i.e. segmented or nonsegmented), were transformed into dummy variables using the “pd.get_dummies” function. The features and target variable were then separated, and feature scaling was performed using the StandardScaler to ensure that all features were on a similar scale. The Random Forest model was performed using the scikit-learn library (Buitinck et al. 2013) in Python (version 3.10.14). The data were randomly split into training (80%) and testing (20%) sets for each iteration of the model training, which was repeated 100 times. During each iteration, feature importance was recorded, and 5-fold cross-validation was conducted to evaluate the model’s performance, utilizing mean squared error (MSE) as the scoring metric. This approach involved dividing the training data into five subsets, training the model on four subsets, and validating it on the remaining subset. This process was repeated five times, with each subset serving as the validation set once, resulting in an average MSE across all folds. Additionally, SHapley Additive exPlanations (SHAP) values (Lundberg 2017) were computed using SHAP TreeExplainer library to quantify the impact of each feature on the target variable. The results, including the mean and standard deviation of feature importance and SHAP values, were compiled into a Pandas DataFrame and saved in an Excel file for further analysis.

## Results

The International Committee on Taxonomy of Viruses currently approves eight primary taxonomic ranks for RNA viruses (realm, kingdom, phylum, class, order, family, genus, and species in descending order) and seven secondary ranks (subrealm, subkingdom, subphylum, subclass, suborder, subfamily, and subgenus) (Simmonds et al. 2024). The realm *Riboviria* includes the kingdom *Orthornavirae*, which comprises RNA viruses encoding an RNA-dependent RNA polymerase (RdRp), and the kingdom *Pararnavirae* that consists of reverse-transcribing viruses that encode a reverse transcriptase. The *Orthornavirae* currently includes four phyla—*Lenarviricota, Pisuviricota, Kitrinoviricota, Duplornaviricota*, and *Negarnaviricota*—that contain animal RNA viruses, and the phylum *Lenarviricota* that likely comprises bacterial, fungal, and protist RNA viruses.

**Figure 1.**
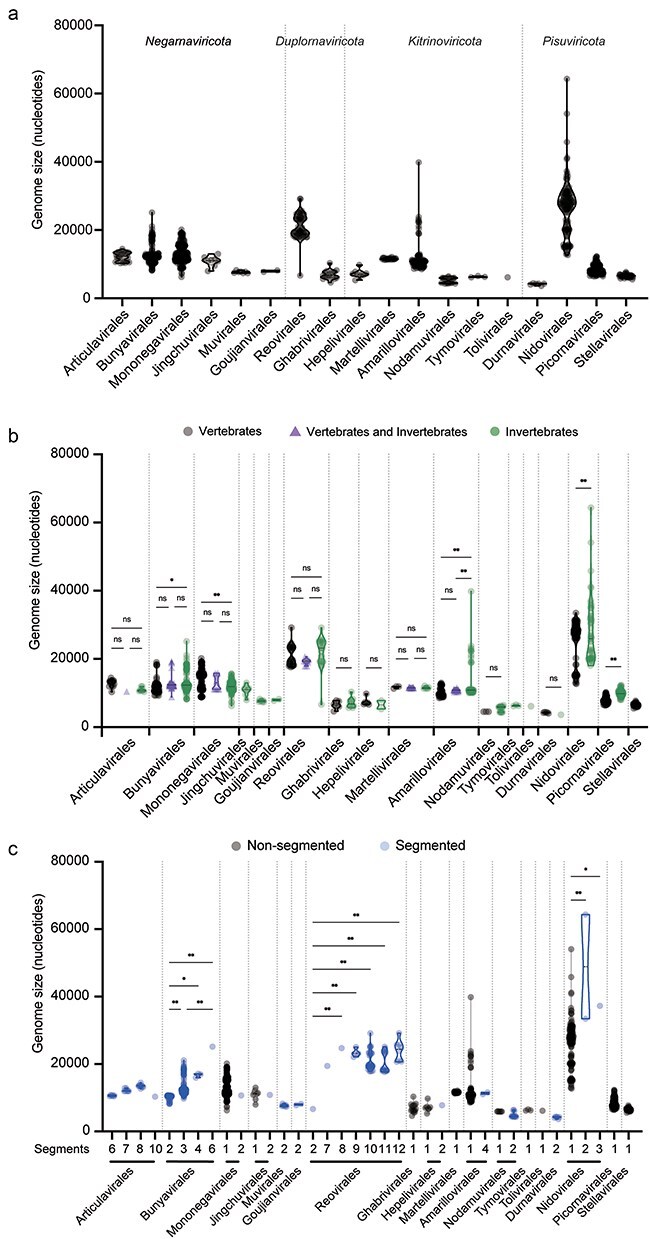
Genome sizes of animal RNA viruses within each viral order. (a) Genome sizes of animal RNA viruses belonging to each order studied. (b) Genome sizes of vertebrate- and invertebrate-associated RNA viruses in each order. Vertebrate-associated RNA viruses, RNA viruses associated with both vertebrates and invertebrates, and invertebrate-associated RNA viruses are shown in black, purple, and green, respectively. (c) Genome sizes of nonsegmented and segmented RNA viruses in each viral order. Nonsegmented and segmented RNA viruses are shown in black and blue, respectively. The number of genome segments is indicated above the name of each viral order (in which “1” denotes a nonsegmented genome). In all cases, truncated violin plots are shown, with the mean values within each viral order compared by using a one-way ANOVA, followed by Šídák’s multiple comparisons test (ns = not significant with *P* > .05; *P < .05; **P < .01).

To reveal the diversity of genome lengths at the level of virus order in the diverse animal-derived RNA viruses found within each of the four *Riboviria* phyla (i.e. *Pisuviricota, Kitrinoviricota, Duplornaviricota*, and *Negarnaviricota*), excluding those associated with plants and other hosts, we compared the size of complete genomes of viruses registered in the Virus Genome Resource (https://www.ncbi.nlm.nih.gov/genomes/GenomesGroup.cgi?taxid=10239&sort=taxonomy). This analysis revealed considerable variation in viral genome sizes between orders (Fig. 1A, Table 1). In the 18 viral orders analyzed here, the *Durnavirales* had the smallest genome sizes (mean of 4148 ± 268.1 nt), while the *Nidovirales* had the largest (mean of 26 178 ± 7 457 nt, but up to 64 336 nt) (Table 1). Indeed, the median and both lower and upper 95% confidence intervals of the mean were the higher in the *Nidovirales* than other orders (median of 27 464 nt, lower 95% confidence interval = 25 002 nt, upper 95% confidence interval = 27 354 nt) (Table 1). The orders *Reovirales* and *Amarillovirales* also contained viruses with large genome sizes (up to 29 174 nt and 39 834 nt, respectively), with the *Amarillovirales* characterized by a highly skewed distribution toward smaller genome sizes and with extreme values at the distribution tails (range of 31 266 nt; from 8568 to 39 834 nt, skewness of 4.094, and kurtosis of 21.67) (Table 1). The *Nidovirales* similarly has a very wide range of genome sizes, spanning 51 632 nt, from 12 704 nt in Equine arteritis virus to 64 336 nt in Crassostrea gigas nidovirus (Fig. 1a, Table 1, Supplementary Table 1).

To determine whether there were differences in the variation in genome size between viral orders, we performed the mean-based Levene’s test of equality of variance (Draper and Hunter 1969), excluding *Goujianvirales* and *Tolivirales*, which only contained two and one viruses, respectively (Table 1). The distance (i.e. absolute deviation) from the mean of each virus genome was calculated for each virus in each order, which were then compared between orders. As expected, the *Nidovirales* had significantly larger mean genome sizes than other orders (*P* < .01) (Supplementary Fig. 1). In addition, the mean genome size of the *Mononegavirales* was significantly larger than that of the *Bunyavirales, Martellivirales*, and *Stellavirales* (*P* = .0184, *P* = .003, *P* < .0001, and *P* < .0001, respectively), while that of the *Reovirales* was significantly larger than that of the *Bunyavirales, Martellivirales, Picornavirales*, and *Stellavirales* (*P* = .0016, *P* = .0001, *P* < .0001, and *P* < .0001, respectively) (Supplementary Fig. 1). There were also significant differences in mean genome sizes between the *Amarillovirales* and *Martellivirales, Amarillovirales* and *Picornavirales*, and *Amarillovirales* and *Stellavirales* (*P* = .0124, *P* = .0016, and *P* = .0008, respectively) (Supplementary Fig. 1). Hence, these results indicate that the genome sizes of animal-derived RNA viruses vary widely both within and between viral orders.

**Figure 2.**
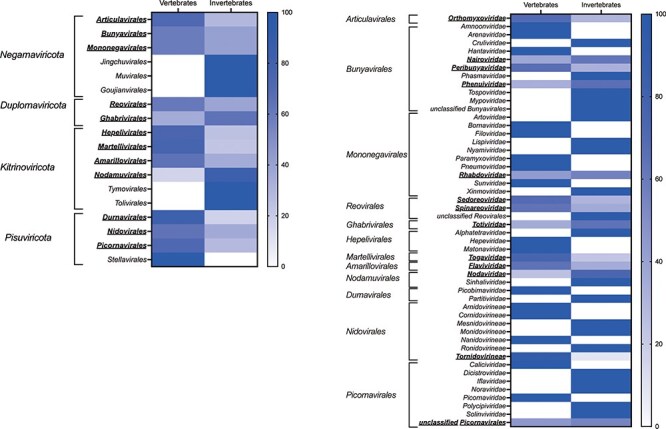
Vertebrate and invertebrate-associated RNA viruses in each viral order and viral family. (a) Heatmap showing the proportion of vertebrate- or invertebrate-associated RNA viruses in each viral order. Underlined bold letters indicate the virus orders that include both vertebrate- and invertebrate-associated viruses. (b) Heatmap showing the proportion of vertebrate- or invertebrate-associated RNA viruses in each virus family in the viral orders that contained both types of virus. Underlined bold letters indicate the virus families that include both vertebrate- and invertebrate-associated viruses.

Since characteristics of viruses are strongly dependent on their host and how they respond to viral infections, it is conceivable that viral genome size could be affected by the major host group they infect. We therefore analyzed whether the animal RNA viruses analyzed here were associated with either vertebrates or invertebrates. Accordingly, some viral orders (*Articulavirales, Bunyavirales, Mononegavirales, Reovirales, Ghabrivirales, Hepelivirales, Martellivirales, Amarillovirales, Nodamuvirales, Durnavirales, Nidovirales*, and *Picornavirales*) contained both vertebrate- and invertebrate-associated RNA viruses (Fig. 2). The same was true at the family level, where *Orthomyxoviridae, Nairoviridae, Peribunyaviridae, Phenuiviridae, Rhabdoviridae, Sedoreoviridae, Spinareoviridae, Totiviridae, Togaviridae, Flaviviridae, Nodaviridae*, and *Tornidovirineae* contained both vertebrate- and invertebrate-associated RNA viruses (Fig. 2).

**Figure 3.**
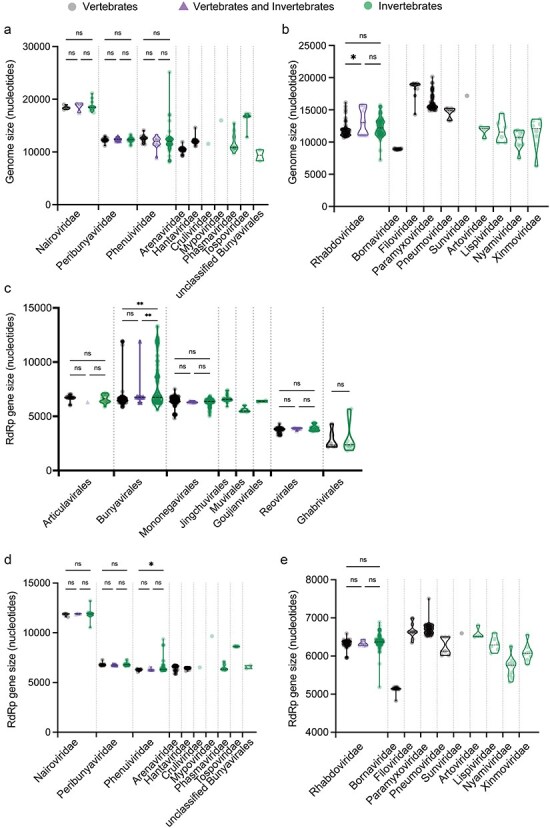
Genome sizes and RdRp gene lengths in animal RNA viruses. (a and b) Genome sizes of RNA viruses in each viral family belonging to the (a) *Bunyavirales* and (b) *Mononegavirales*. Vertebrate-associated RNA viruses, both vertebrate- and invertebrate-associated RNA viruses, and invertebrate-associated RNA viruses are shown in black, purple, and green, respectively. (c) Size of the RdRp gene in each viral order within the *Negarnaviricota* and *Duplornaviricota*. Vertebrate-associated RNA viruses, RNA viruses associated with both vertebrates and invertebrates, and invertebrate-associated RNA viruses are shown in black, purple, and green, respectively. (d and e) Size of the RdRp gene in each viral family within the *Bunyavirales* (d) and *Mononegavirales* (e). Vertebrate-associated RNA viruses, both vertebrate- and invertebrate-associated RNA viruses, and invertebrate-associated RNA viruses are shown in black, purple, and green, respectively. In all cases, mean values within each viral family were compared by using a one-way ANOVA, followed by Šídák’s multiple comparisons test (ns = not significant with *P* > .05; *P < .05; **P < .01).

We similarly compared the genome sizes of the vertebrate- and invertebrate-associated RNA viruses within each virus order. Notably, in the *Bunyavirales, Amarillovirales, Nidovirales*, and *Picornavirales*, invertebrate-associated viruses had significantly larger genome sizes than vertebrate viruses (*P* = .0135, *P* < .0001, *P* < .0001, and *P* < 0.0001, respectively) (Fig. 1b). Conversely, in *Mononegavirales*, the genome size of vertebrate-associated viruses was significantly larger than that of invertebrate viruses (*P* < .0001) (Fig. 1b). This was the only group in which vertebrate viruses were longer than those in invertebrates. Nonsignificant differences between vertebrate and invertebrate viruses were observed in the remaining interorder comparisons. Similarly, we compared the genome sizes of vertebrate- and invertebrate-associated viruses within each virus family in those orders that showed differences in genome size between these major host groups. All viruses in the *Amarillovirales* analyzed here (unclassified viruses excluded) belonged to *Flaviviridae*, within which the invertebrate-associated viruses had significantly larger genome sizes than those associated with vertebrates (*P* < .0001) (Supplementary  Fig. 2). In the *Nidovirales*, the *Arnidovirineae*, which is composed solely of vertebrate-associated viruses, had smaller genomes than other families in that order that are composed exclusively of invertebrate-associated viruses, while in the *Tornidovirineae*, there were no significant differences in the size of genome between vertebrate- and invertebrate-associated viruses (*P* = .709) (Supplementary  Fig. 2). In the *Picornavirales*, *Caliciviridae* and *Picornaviridae*, which are composed exclusively of vertebrate-associated viruses, had smaller genomes than other families in that order that are composed exclusively of invertebrate-associated viruses (Supplementary Fig. 2). In the *Phenuiviridae* (*Bunyavirales*) and *Rhabdoviridae* (*Mononegavirales*), there were no significant differences in the genome sizes of vertebrate- and invertebrate-associated viruses (*P* = .9186 and *P* = .0627, respectively) (Fig. 3a and 3b). Finally, in the *Mononegavirales*, the *Filoviridae, Paramyxoviridae, Pneumoviridae*, and *Sunviridae*, which exclusively comprise vertebrate viruses, had larger genomes than other families in that order that only contained invertebrate viruses (Fig. 3b).

To determine whether the differences in genome size may be associated with differences in the size of the gene that dictates virus replication, we compared the sizes of gene encoding the RNA-dependent RNA polymerase (RdRp)—a defining characteristic of RNA viruses—within each viral order. In some positive-stranded RNA viruses (i.e. the phyla *Pisuviricota* and *Kitrinoviricota* and hence including the nidoviruses), the RdRp is part of a larger polyprotein, making an accurate comparison of the gene encoding the RdRp difficult. For simplicity of analysis, we therefore only considered viruses from the *Negarnaviricota* and *Duplornaviricota* where the start and stop codons for the RdRp gene can be clearly defined. This revealed that the size of the RdRp gene in invertebrate-associated viruses was significantly larger than that in vertebrate viruses in the *Bunyavirales* (*P* < .0001), while there were no significant differences in the *Mononegavirales, Articulavirales, Reovirales*, and *Ghabrivirales* (Fig. 3c). To examine these differences in more detail, we compared the genome size and the gene size encoding RdRp within each viral family in the *Bunyavirales* and *Mononegavirales*. In the *Phenuiviridae* (*Bunyavirales*), invertebrate-associated RNA viruses have significantly larger RdRp genes than vertebrate viruses (*P* = .0183) (Fig. 3a and 3d) and slightly larger genomes than vertebrate viruses, although this difference was not significant (*P* = .9186). In contrast, in the *Rhabdoviridae* (*Mononegavirales*), there were no significant differences in the size of genome and the RdRp gene between invertebrate- and vertebrate-associated viruses (*P* = .0627 and *P* = .0551, respectively) (Fig. 3b and 3e). However, there was a strong correlation between genome size and the size of the gene encoding RdRp in several groups (Supplementary fig. 3). In the *Bunyavirales*, there was a strong correlation in both vertebrate- (*R*^2^ = 0.6469, *P* < .0001) and invertebrate-associated viruses (*R*^2^ = 0.7284, *P* < .0001) (Supplementary Fig. 3), while in the *Mononegavirales*, there was a strong correlation in vertebrate-associated viruses (*R*^2^ = 0.5946, *P* < .0001), although it was less pronounced in invertebrate-associated viruses (*R*^2^ = 0.1566, *P* < .0001) (Supplementary Fig. 3). Hence, these results suggest that the increased gene size of RdRp may be one factor that contributes to genome size expansion, although this differs between virus orders and families.

We also compared the genome sizes between segmented and nonsegmented viruses within each viral order. Some orders of RNA viruses contain both segmented and nonsegmented viruses (i.e. *Mononegavirales, Jingchuvirales, Hepelivirales, Amarillovirales, Nodamuvirales*, and *Nidovirales*), while others only contain segmented viruses (i.e. *Articulavirales, Bunyavirales, Muvirales, Goujianvirales, Reovirales*, and *Durnavirales*). The *Negarnaviricota* contains two subphyla—*Haploviricotina* (e.g. *Mononegavirales* and *Jingchuvirales*)—that primarily comprise viruses with nonsegmented genomes, and *Polyploviricotina* (e.g. *Articulavirales*, and *Bunyavirales*) that largely consists of viruses with segmented genomes (Kuhn et al. 2019, Siddell et al. 2019, Koonin et al. 2020). In addition, the *Duplornaviricota* has viral orders that contain only segmented or nonsegmented RNA viruses. Notably, segmented viruses within the *Nidovirales* (of which there are three—Longidorus elongatus nidovirus, Hydra vulgaris nidovirus 2, and Crassostrea gigas nidovirus) had significantly larger genome sizes than nonsegmented viruses (Fig. 1c; Supplementary Table 1). Conversely, there were no significant differences in the size of the segmented and nonsegmented genomes in the *Mononegavirales, Jingchuvirales, Hepelivirales, Amarillovirales*, and *Nodamuvirales* (Fig. 1c). Hence, these results again suggest that whether viral genome segmentation contributes to an increase in viral genome size depends on the taxonomic group.

Next, we compared genome sizes between viruses with differing numbers of segments. In the *Bunyavirales*, viruses with three or more genome segments had significantly larger genome sizes than viruses with two segments (*P* < .05) (Fig. 1c), while viruses with six segments had significantly larger genome sizes than viruses with three segments (*P* = .0039) (Fig. 1c). In the case of the *Reovirales*, Nephila clavipes virus 6, which has two genome segments, had a significantly smaller genome than viruses with 8, 9, 10, 11, or 12 segments (*P* = .0018, *P* < .0001, *P* = .0003, *P* = .0005, and *P* < .0001, respectively), while there was no significant difference in genome size among viruses with more than seven segments (*P* > .3) (Fig. 1c). In the *Articulavirales*, there were no significant differences in genome size among viruses possessing any number of genome segments (*P* > .9) (Fig. 1c). For the *Articulavirales*, we also examined whether the average size of segments differed among viruses possessing 6, 7, 8, or 10 genome segments. Accordingly, viruses with eight segments had significantly smaller average segment sizes than those with six segments but not those with seven segments (*P* < .0001 and *P* = .0805, respectively), while the average size of segments in Tilapia lake virus that has 10 genome segments was significantly smaller than that of viruses that possessed fewer segments (*P* < .0001 in all cases) (Supplementary  Fig. 4). Hence, genome sizes are maintained despite differences in the number of genome segments.

**Figure 4.**
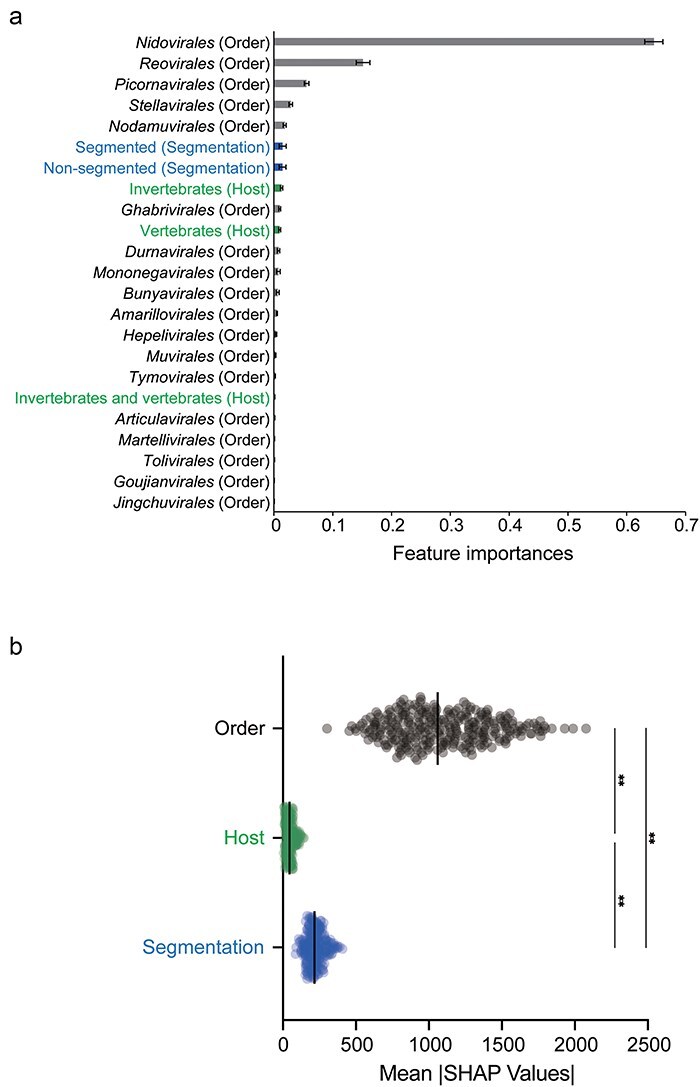
Statistical importance of viral order, host, and segmented genome in determining the genome sizes of animal RNA viruses. (a) Mean feature importance in relation to genome size. Data are shown as the mean of 100 replications. Error bars indicate the standard deviation. The features included in each qualitative trait’s groups (i.e. virus order, host, and segmentation group) are shown in gray, green, and blue, respectively. Each feature is ordered from top to bottom based on its mean importance, with the highest at the top. (b) Evaluating contributions of qualitative traits to genome size using mean SHAP values. The mean of the sum of the absolute mean SHAP values for all samples (data points) was calculated for each of the qualitative traits: viral order, host, and segmentation. The mean values were compared by using a one-way ANOVA, followed by Tukey’s multiple comparisons test (**P < .01).

We also examined patterns of genome segmented between the vertebrate- and invertebrate-associated RNA viruses in each viral order (the exceptions were the *Jingchuvirales, Muvirales*, and *Goujianvirales* that only contained invertebrate-associated viruses). In our analysis, 188 invertebrate-associated viruses contained segmented genomes compared to 184 vertebrate viruses. In the *Articulavirales*, vertebrate RNA viruses had more segments than invertebrate RNA viruses (Supplementary Fig. 5). In contrast, in the *Bunyavirales* and *Reovirales*, invertebrate RNA viruses had more segments than vertebrate RNA viruses (Supplementary Fig. 5). In the *Mononegavirales, Hepelivirales, Amarillovirales*, and *Nidovirales*, segmented genomes were only found in invertebrate-associated RNA viruses (Supplementary Fig. 5), while in the *Nodamuvirales*, nonsegmented genomes were only found in invertebrate RNA viruses. Although they were not included in our analysis, bisegmented nidoviruses have recently been found in some vertebrates (Lauber et al. 2024). Lastly, in the *Durnavirales*, all viruses had bisegmented genomes, such that there were no differences in the number of segments between invertebrate- and vertebrate-associated RNA viruses (Supplementary Fig. 5).

Finally, we analyzed the association between the genome length of segmented and nonsegmented viruses, and with that of the RdRp gene, in the *Negarnaviricota* and *Duplornaviricota*. In the case of the *Negarnaviricota*, there was no significant difference in genome size between viruses with segmented and nonsegmented genomes (*P* = .2525) (Supplementary Fig. 6), although the RdRp gene was significantly larger in viruses with segmented than nonsegmented genomes (*P* < .0001). Similar results were observed even if the RdRp gene of the *Articulavirales* was assumed to represent PB1 only (*P* = .2512 and *P* = .0008 for genome size and RdRp gene size, respectively) (Supplementary Fig. 7C). In contrast, in the *Duplornaviricota*, genome size was significantly larger in segmented than nonsegmented genome viruses (*P* = .0032), while there was no significant difference in size of the RdRp gene between segmented and nonsegmented viruses (*P* = .1859) (Supplementary Fig. 6).

To assess the relative importance of individual features—host (i.e. invertebrate or/and vertebrate), viral order (18 viral orders), and genome segmentation (i.e. segmented or nonsegmented)—in determining virus genome size, we used a Random Forest regression model to analyze the genome size data to be linked to each information. We first calculated “feature importance” which provides information on the relative significance of each covariate in building the model. This analysis revealed that viral order (particularly the *Nidovirales, Reovirales, Picornavirales, Stellavirales*, and *Nodamuvirales*) was an important feature in determining genome size (Fig. 4a). In the other virus orders, the feature importance of viral order was similar to or lower than that of the genome segmentation (i.e. segmented or nonsegmented) or host group (i.e. invertebrate and/or vertebrate) (Fig. 4a). To evaluate the contribution of each feature (viral order, host, and segmentation) to genome size and to assess how they affected the prediction results, we calculated the sum of the absolute mean SHapley Additive exPlanations (SHAP) values for each of these features. We then compared the mean of these sums across all data points. This revealed that viral order was significantly higher in value than both segmentation and animal host (*P *< .0001 for both) (Fig. 4b), indicating that it contributes most to genome size. Overall, therefore, viral order has a greater impact in determining the genome size of RNA viruses that genome segmentation or major animal host.

## Discussion

We performed a detailed analysis of the variation in genome sizes, and its determinants, within and among different orders and families of animal RNA viruses. Our results indicate that the genome sizes of animal RNA viruses exhibit significant variation both within and between different viral orders. In particular, the *Nidovirales*, which includes the coronaviruses, had a far greater variation in genome size than other viral orders. The larger genome sizes for at least some viruses in this group are likely explained by the presence of the ExoN domain that reduces the mutation rate and hence enables genomes to expand in size without accumulating excessive deleterious mutations. Indeed, it is notable that the smallest nidovirus—Equine arteritis virus at 12 704 nt—lacks an ExoN domain (Saberi et al. 2018). However, it is also of note that viral genomes of comparable size to those of the *Nidovirales* have recently been detected in the *Flaviviridae*, which, however, seem to lack a detectable ExoN domain (Petrone et al. 2024). Hence, there might be other evolutionary solutions for RNA viruses to overcome the notional error threshold. In this context, it is notable that the genomes of invertebrate-associated viruses were significantly larger than those associated with vertebrates in both the *Nidoviridae* and the *Amarillovirales* (i.e. *Flaviviridae*). Thus, a better understanding of the mechanisms by which large viruses overcome mutational burden might be found in further studies of invertebrate-associated viruses.

We also found a complex relationship between RNA virus genome size and major animal host type and pattern of genome segmentation. In most cases, there were no significant differences in the genome sizes of vertebrate or invertebrate viruses, although when there were differences in invertebrate viruses tended to be associated with longer genomes (i.e. the *Bunyavirales, Amarillovirales, Nidovirales*, and *Picornavirales*) with the *Mononegavirales* showing the opposite pattern. Although the reasons for the increased genomes in the vertebrate *Mononegavirales* are unclear, it is striking that this size increase was associated with multiple families (i.e. the *Filoviridae, Paramyxoviridae, Pneumoviridae*, and *Sunviridae*) that only contain vertebrate viruses. In addition, it is notable that the longest invertebrate-associated picornavirus in the order *Picornavirales* (12 333 nt) did not exceed the smallest vertebrate-associated nidovirus in the order *Nidovirales* (12 704 nt), highlighting the impact of virus order. With these uncertainties notwithstanding, it is striking that the viruses of vertebrates do not follow their hosts in generally being larger and more complex than those of invertebrates.

Any association between genome segmentation and genome size similarly seemed to depend on viral order. In the case of positive-stranded RNA viruses, within which genome segmentation has evolved multiple times, this trait was associated with larger genomes in the *Nidovirales*, while there was no difference among the *Amarillovirales, Hepelivirales*, and *Nodamuvirales*. In the case of double-stranded RNA viruses and negative-stranded RNA viruses, genome segmentation was associated with larger genomes in the *Duplornaviricota*, while there was no significant difference in the *Negarnaviricota*. Interestingly, segmented genomes were only found in invertebrate-associated RNA viruses in the *Mononegavirales, Hepelivirales, Amarillovirales*, and *Nidovirales*. Recently, bisegmented nidovirus genomes have been identified in vertebrates (Lauber et al. 2024), although the genome originally annotated as monopartite in GenBank (accession MK611985) has not yet been reannotated. There is currently no clear explanation for why genome segmentation evolved in RNA viruses, with theories including a means to reduce mutational load or to better control gene expression (Holmes 2009), or why the number of segments varies so markedly among virus groups. However, it is the case that the more virus genomes are sequenced from diverse animal taxa, so genome segmentation appears to be a flexible evolutionary characteristic, appearing in diverse phylogenetic locations, rather than one that defines families of RNA viruses (Shi et al. 2018, Hou et al. 2025).

In addition to the low fidelity of RNA replication, the genome sizes of RNA viruses could be constrained by structural factors at the nucleotide and protein levels. RNA viruses utilize RNA secondary structures, such as stem-loops or hairpin structures, to efficiently compact genetic information, allowing them to perform multiple functions with limited genomic material. For example, dengue viruses have stem-loops in the 3ʹ UTR for polymerase recognition (Villorodo et al. 2015), and polioviruses contain a hairpin structure in the 5ʹ UTR that facilitates efficient protein translation (Simoes and Sarnow 1991), while avian influenza viruses have stem-loop structures that enable the insertion of basic amino acid codons and contribute to the emergence of highly pathogenic strains (Kida et al. 2021). Additionally, some RNA viruses synthesize large polyproteins, enabling a single precursor to generate multiple proteins (including the RdRp) within a single open-reading frame, thereby optimizing genome utilization. Protein structure and size also play a key role in genome replication and immune evasion. However, in this context, it is important to note that our study only considered nucleotide-level factors, specifically genome size and RdRp gene size, with the exclusion of structural factors. From this analysis, and in the particular virus groups examined, we determined that RdRp gene size may be correlated with genome size, although the relationship is complex. This observation is in accord with a previous study which showed that RNA viruses with larger genomes tend to have relatively large polymerase proteins, potentially reflecting higher replication fidelity (Belshaw et al. 2008), although this is yet to be experimentally validated. More broadly, these findings highlight the potential for combining protein structure prediction with experimental functional evaluation to provide deeper insights into the evolution of genome size in RNA viruses.

It has been proposed that various aspects of virus morphology, such as the size and shape of viral particles, impact aspects of virus assembly and in so doing constrain the sizes of virus genomes. For example, while influenza A and B viruses have eight-segment genomes and influenza C and D viruses have seven-segment genomes, all can incorporate eight RNA segments within their particles (Noda et al. 2006, 2012, Nakatsu et al. 2016, 2018). Our results show that in the *Articulavirales* (that includes influenza viruses), six segment viruses have significantly larger average segment sizes than seven- and eight-segment viruses, although there is no association between segment number and genome size. Hence, there may have been selection for an optimal number and size of segments within the range that can be stably packaged into particles, which, however, fall within an upper limit on overall genome size. In support of this, Tilapia lake virus has a 10-segment genome, but is slightly smaller in size than *Articulavirales* with eight-segment genomes (although the difference is not significant) and with significantly smaller genome segments. How the segments of Tilapia lake virus are packaged is unknown, but it is possible that by reducing segment sizes, Tilapia lake virus might overcome the constraints on the number of RNA segments for stable packaging. Likewise, in the *Reovirales*, there were no significant differences in genome size among viruses containing between 7 and 12 segments, suggesting that there are overall constraints in genome size irrespective of the number of genome segments. However, genome size and capsid volume are not proportional in RNA viruses compared to DNA viruses (Chaudhari et al. 2021). For example, the capsid volume of RNA viruses can vary by a factor of ∼1000, while their genome size varies by only ∼10 fold (Chaudhari et al. 2021) although, importantly, this does not consider the possibility that the RNA virus genome adopts secondary structures that require less space than a linear molecule. Since the morphological characteristics or mechanics of packaging of the increasingly large number of viruses discovered through metagenomic analysis are not yet well understood, it is evident that a better biophysical characterization of newly discovered viruses is warranted.

A common theory in the evolutionary biology of cellular organisms is that there are important historical—i.e. phylogenetic—constraints on adaptive flexibility that hinder the evolution of optimal adaptive solutions (Gould and Lewontin 1979). Hence, organismal evolution is contingent on the traits inherited from ancestors, and no organism exists in a completely adaptively optimized condition (as such an organism would live forever and have infinite off-spring). These constraints can hinder evolution in other directions. For example, the number of cervical vertebrae in mammals (typically seven) is tightly constrained by genetic interactions during development (Narita and Kuratani 2005, Galis et al. 2006). As our Random Forest regression analysis indicated that RNA virus order (i.e. taxonomic position) has a greater impact on virus genome size than the major host group or pattern of segmentation, it is possible that this theory can be extended to viruses. Hence, the effect of the host on genome size needs to be understood for each viral order, which has inherited a particular set of virological traits.

An important limitation of our study is the very high number of undiscovered viruses, which will number many millions, especially in invertebrate taxa that have only been poorly sampled to date. Recently, metagenomic analyses of various invertebrates have detected viral genomes that greatly extend our current understanding of RNA virus genomes and their evolution (Neuman et al. 2024, Petrone et al. 2024). It is therefore conceivable that viral genome sizes will continue to expand with greater sampling and challenge the observations made here. In addition, there remains controversy regarding the viral taxonomic ranks above the phyla level or order level (Simmonds et al. 2023), limiting the scope of evolutionary discussions in this study in the kingdom *Orthornavirae* in the realm *Riboviria*. The discovery of more novel viruses from diverse hosts will not only help to fill gaps in virus taxonomy but also provide a better understanding of the evolution of genome size of RNA viruses.

## Supplementary Material

veaf005_Supp

## Data Availability

The datasets used in this study are provided in Supplementary Tables 1 and 2.
