# Supplementary material for: Genome sizes of animal RNA viruses reflect phylogenetic constraints
Source: Virus Evol. 2025 Jan 24;11(1):veaf005. doi: 10.1093/ve/veaf005 (PMC11792653; doi:10.1093/ve/veaf005)
Supplement: veaf005_Supp [file veaf005_supp.zip › suppl_data/Takada.Supplementary Figures.pdf]

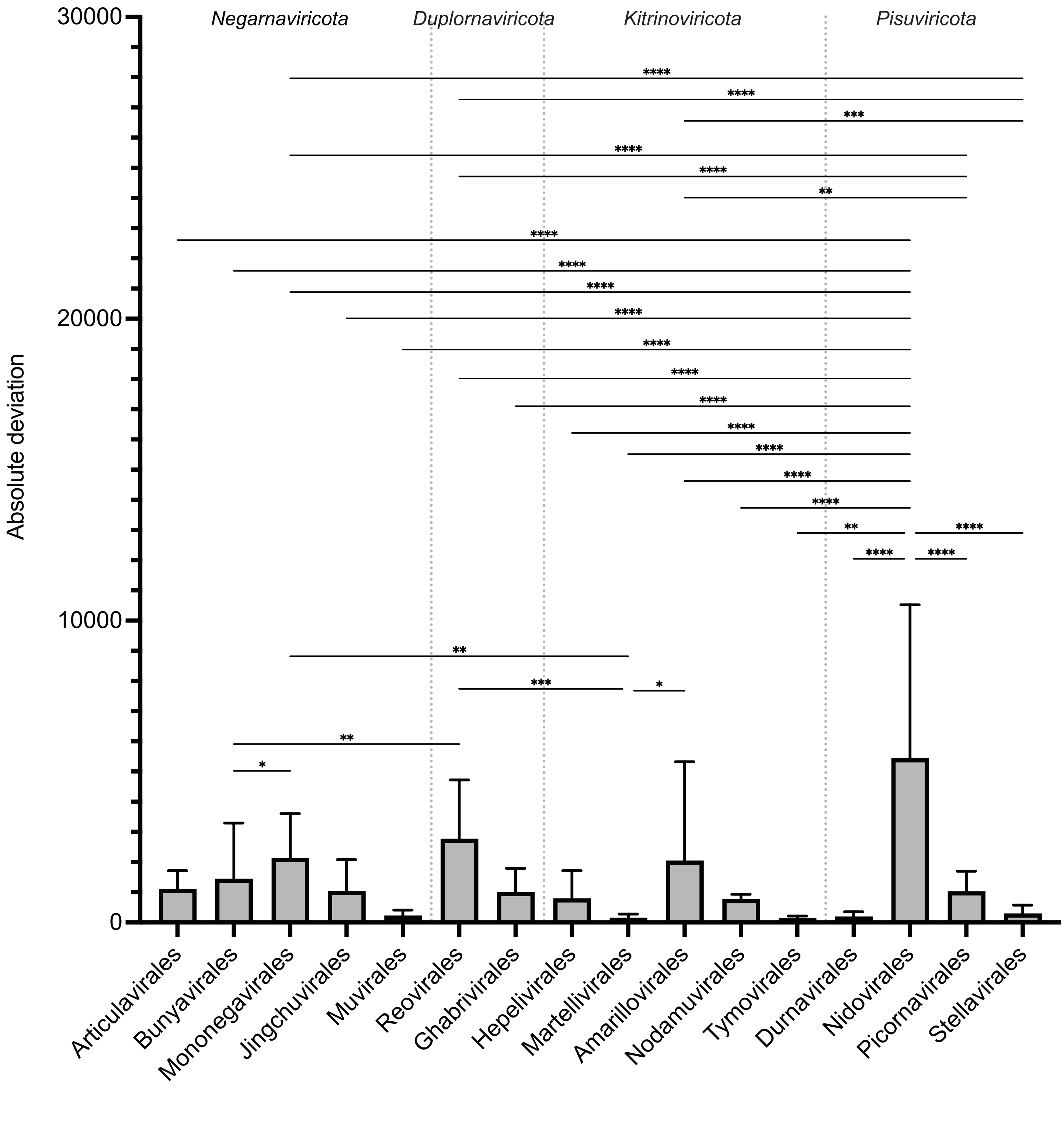

Supplementary Figure 1

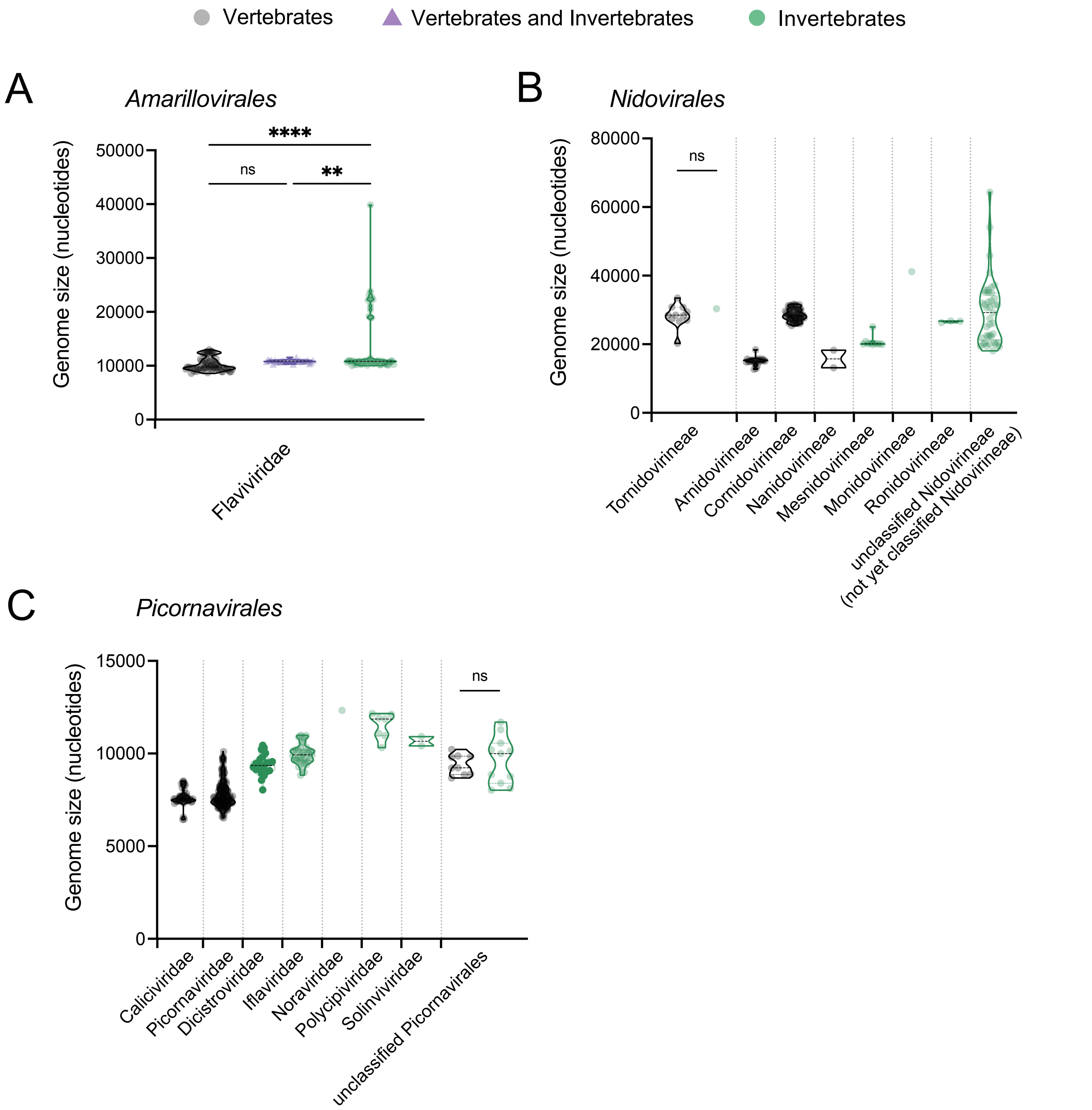

Supplementary Figure 2

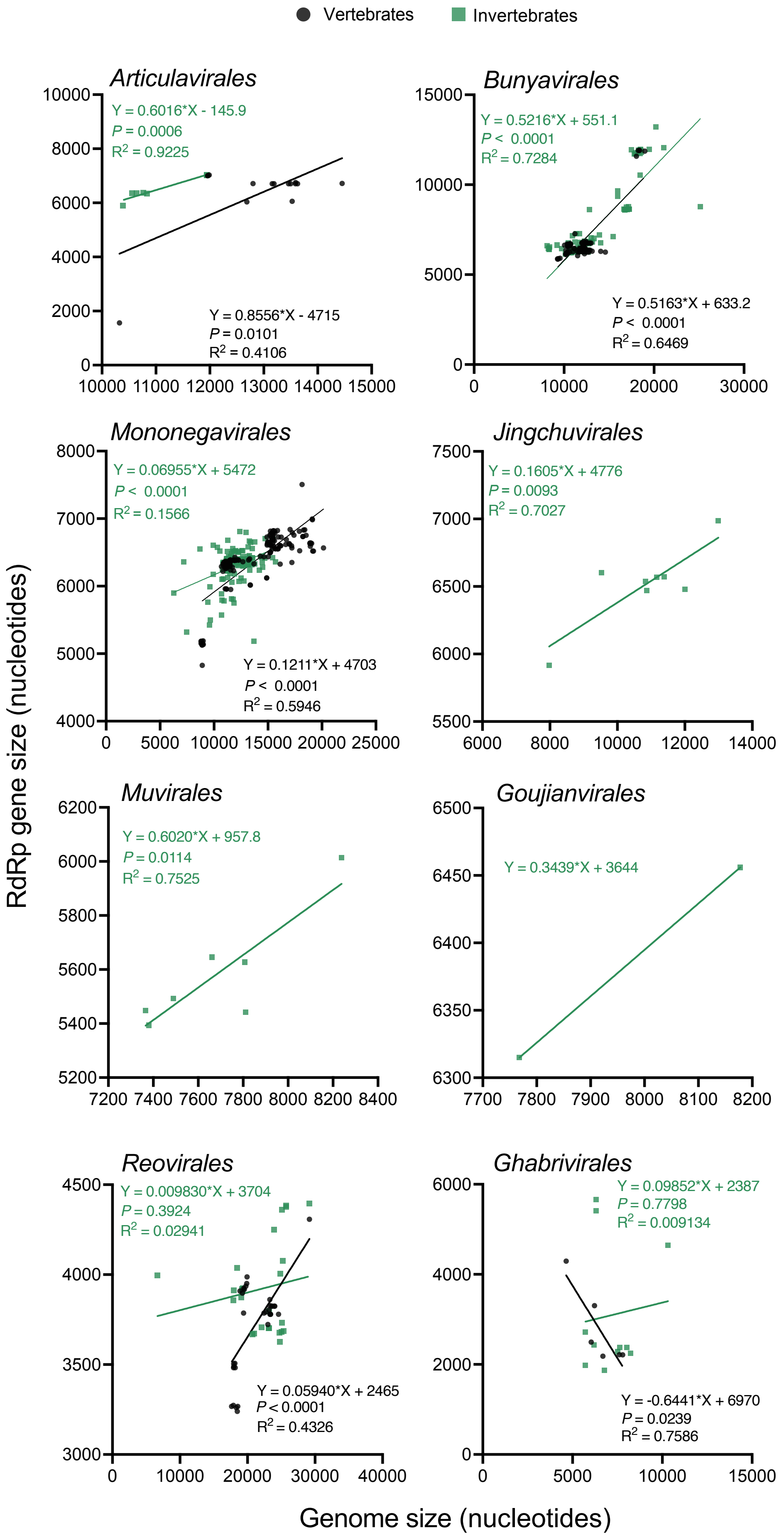

Supplementary Figure 3

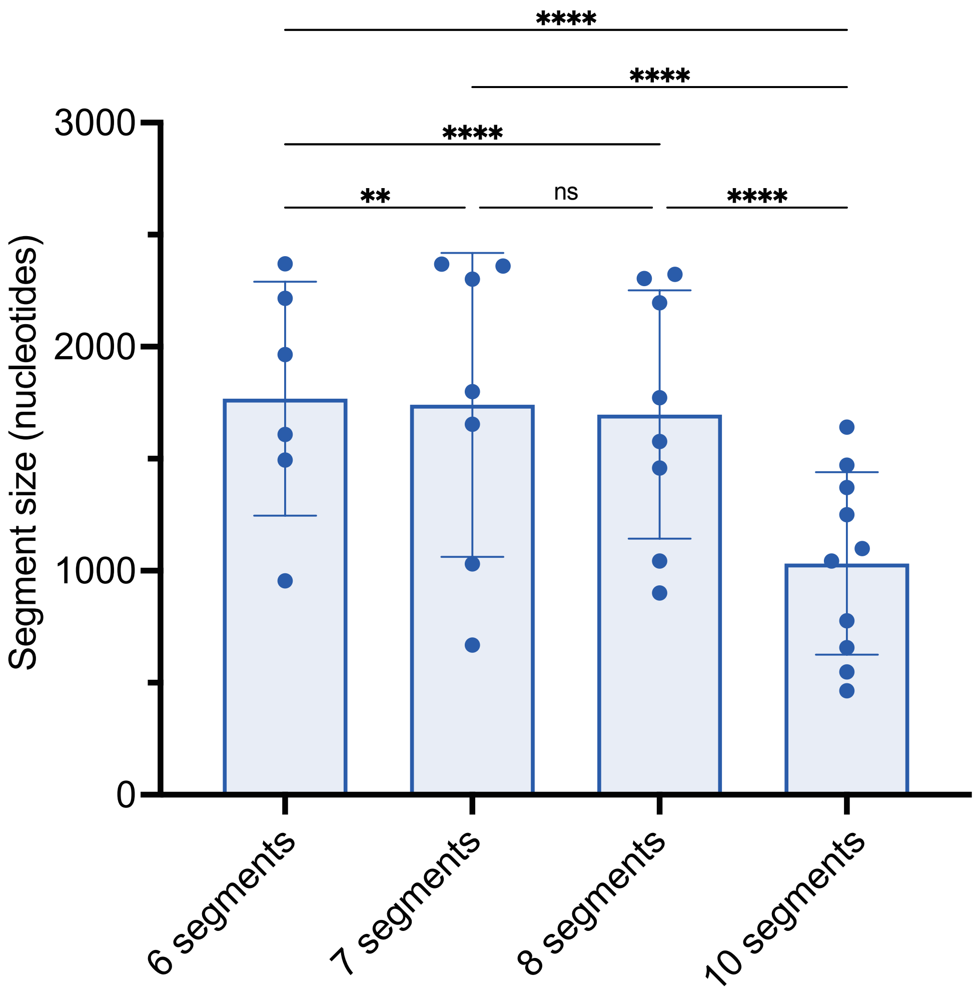

Supplementary Figure 4

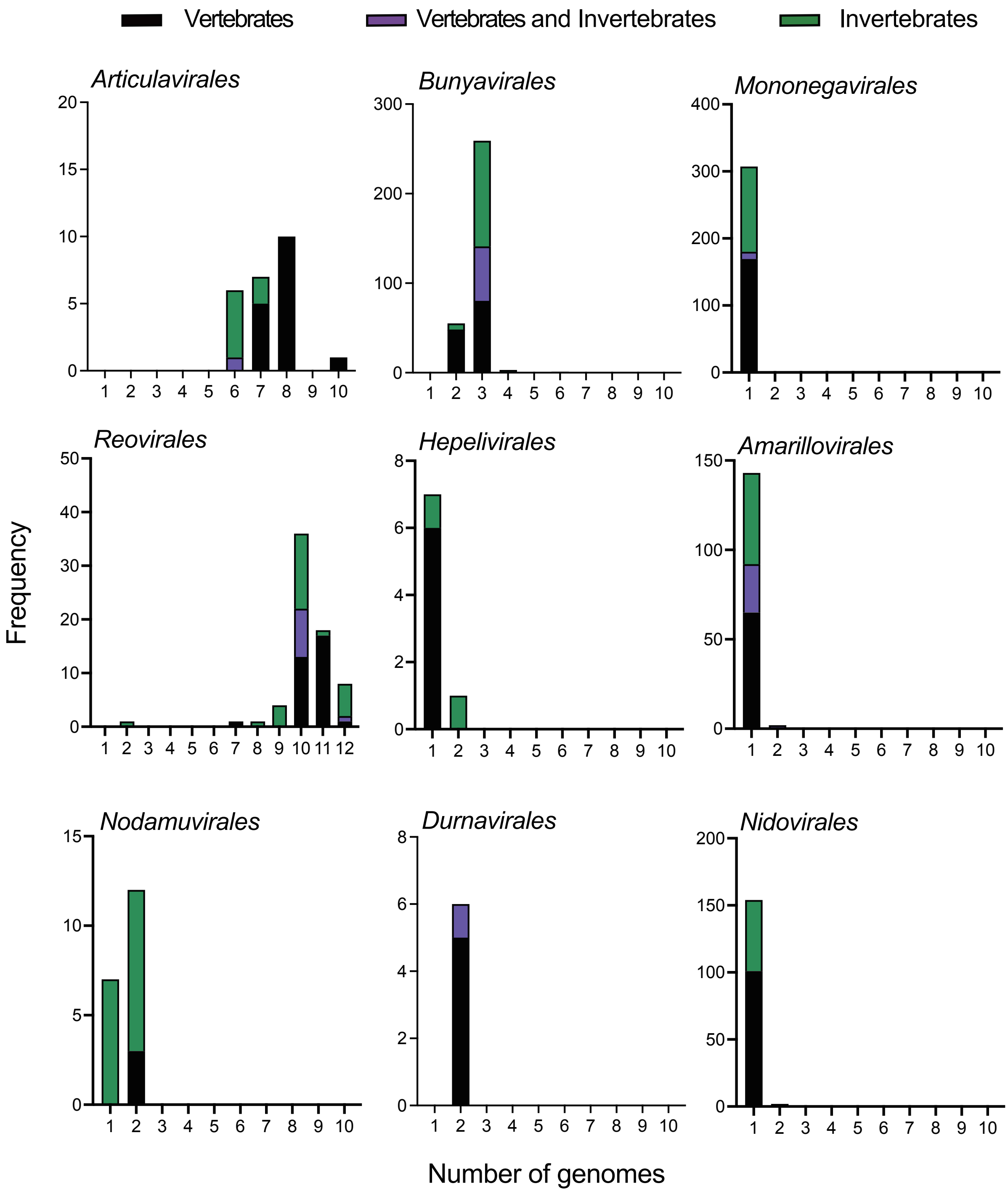

Supplementary Figure 5

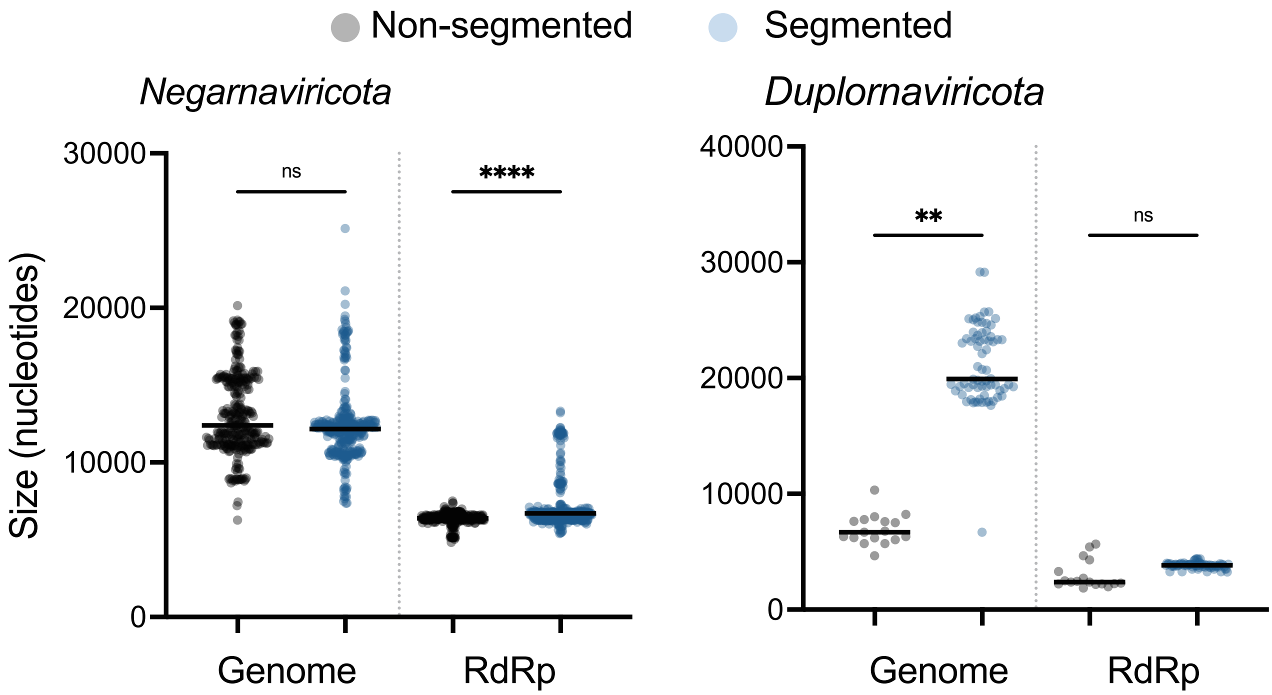

Supplementary Figure 6

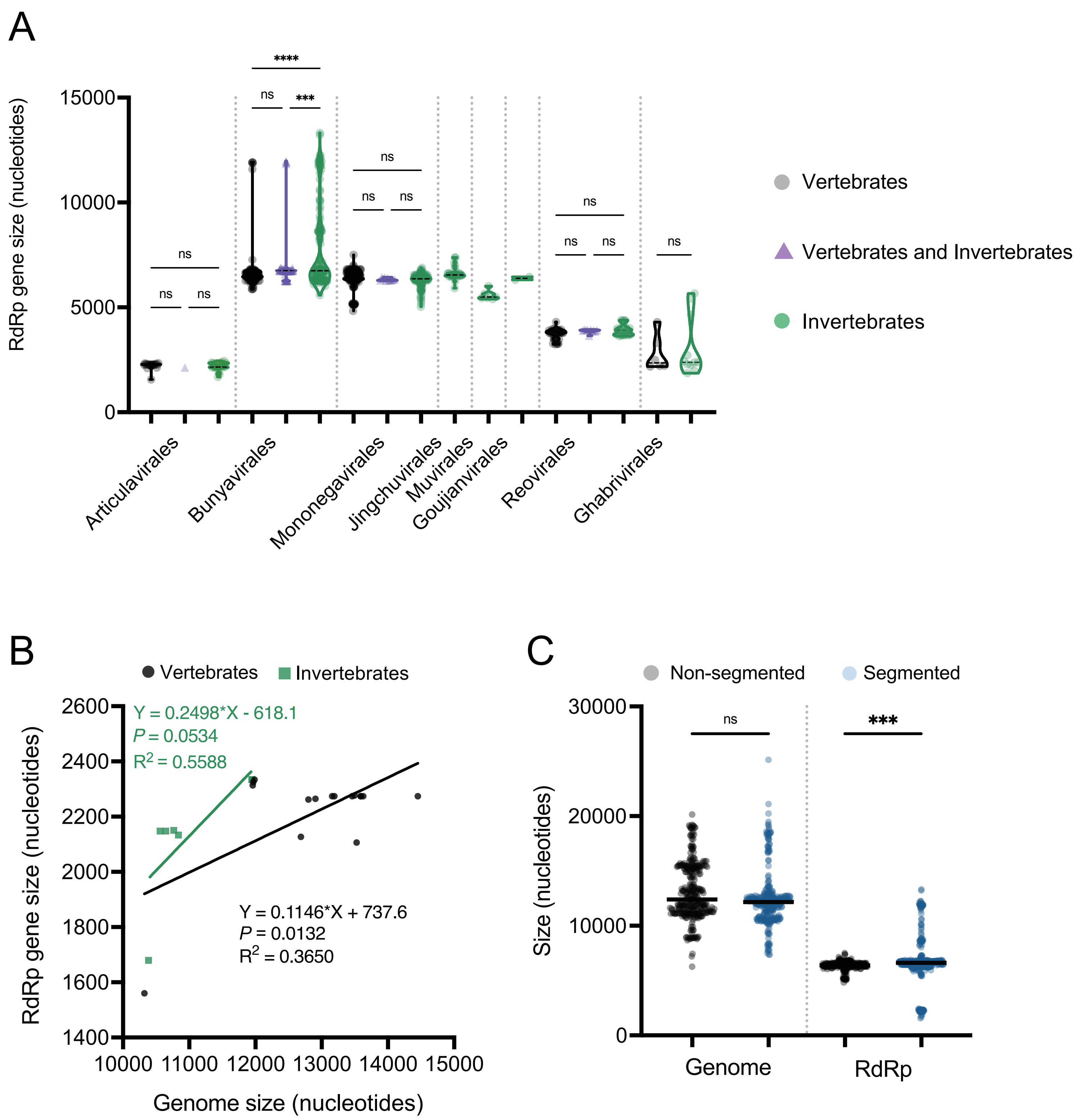

Supplementary Figure 7
